# Knowledge, self-efficacy, satisfaction and self-confidence of nursing students for stoma care using active learning methodologies: a quasi-experimental study of three arms

**DOI:** 10.17533/udea.iee.v43n2e06

**Published:** 2025-07-23

**Authors:** Karima Abselam-Ali, Concepción Capilla-Díaz, Rocío Reina-Prego, María Elena Parra-González

**Affiliations:** 1 Nurse, Ph.D. student. Email: karimaabselam@correo.ugr.es r.reinaprego@ugr.es. https://orcid.org/0000-0003-1126-1364 University Hospital of Ceuta Spain karimaabselam@correo.ugr.es; 2 Nurse, Ph.D. Permanent Lecturer Email: conchicd@ugr.es Corresponding author. https://orcid.org/0000-0002-3361-8718 Universidad de Granada Spain conchicd@ugr.es; 3 Nurse, Ph.D. Junior Lecturer. Email: r.reinaprego@ugr.es. https://orcid.org/0000-0003-1604-2806 Universidad de Granada Spain r.reinaprego@ugr.es; 4 English primary-school teacher, Social and Cultural Anthropology. Senior Lecturer Email: elenaparra@ugr.es. https://orcid.org/0000-0002-6918-9126 Universidad de Granada Spain elenaparra@ugr.es; 5 University Hospital of Ceuta, Ceuta, Spain University Hospital of Ceuta University Hospital of Ceuta Ceuta Spain; 6 Instituto de Investigación Biosanitaria (ibs.GRANADA) Instituto de Investigación Biosanitaria Instituto de Investigación Biosanitaria (ibs.GRANADA) Spain; 7 Department of Nursing, Faculty of Health Sciences, University of Granada, Spain Universidad de Granada Department of Nursing Faculty of Health Sciences University of Granada Spain; 8 CTS-436 PATH Research Group CTS-436 PATH Research Group CTS-436 PATH Research Group Spain; 9 Department of Research Methods and Diagnosis in Education, University of Granada, Spain Universidad de Granada Department of Research Methods and Diagnosis in Education University of Granada Spain

**Keywords:** active learning, nursing education, simulation, surgical stomas, virtual reality., aprendizaje activo, educación en enfermería, simulación, estomas quirúrgicos, realidad virtual., aprendizado ativo, educação em enfermagem, simulação, estomas cirúrgicos, realidade virtual.

## Abstract

**Objective.:**

To assess knowledge about stoma care in relation to the level of learning methods and to measure students' self-confidence, satisfaction and self-efficacy with the training received using different learning methods including simulation.

**Methods.:**

A quasi-experimental study of three arms, with pretest and post-test design in third-year nursing students during the academic year 2022-2023. Group A received traditional teaching methods and low-fidelity simulation, group B received traditional teaching methods and high-fidelity simulation, and group C received active learning methods and high-fidelity simulation.

**Results.:**

Significant differences were found in 14 of the 15 NOC indicators measured between groups. Self-efficacy showed significant differences between the different groups (*p*<0.001) after the intervention. For satisfaction and self-confidence, there were significant differences (*p*<0.001) observed between the different fidelity groups).

**Conclusion.:**

This study found that simulation improved the level of knowledge regarding ostomies and had a positive impact on satisfaction and self-confidence in all three intervention groups, with significantly higher results among participants in Group C (active learning methods and high-fidelity simulation).

## Resumen

**Objetivo.:**

Evaluar los conocimientos sobre ostomías en relación con el nivel de los métodos de aprendizaje y medir la autoconfianza, satisfacción y autoeficacia de los estudiantes con la formación recibida usando diferentes métodos de aprendizaje, incluida la simulación.

**Métodos.:**

Estudio cuasiexperimental de tres brazos, con diseño pretest y postest en estudiantes de tercer curso de enfermería durante el curso 2022-2023. El grupo A recibió métodos tradicionales de enseñanza y simulación de baja fidelidad, el grupo B recibió métodos tradicionales de enseñanza y simulación de alta fidelidad, y el grupo C recibió métodos activos de aprendizaje y simulación de alta fidelidad.

**Resultados.:**

Se encontraron diferencias significativas en 14 de los 15 indicadores NOC medidos entre los grupos. Tras la intervención la autoeficacia mostró diferencias significativas entre los distintos grupos (*p*<0.001). En cuanto a la satisfacción y la autoconfianza, también se observaron diferencias significativas (*p*<0.001).

**Conclusiones.:**

En este estudio se encontró que la simulación mejoró el nivel de conocimientos en relación con las ostomías, y tuvo un impacto positivo en la satisfacción y la autoconfianza en los tres grupos de intervención, siendo mucho más altos los resultados en los participantes del grupo C (métodos de aprendizaje activo y simulación de alta fidelidad).

## Resumo

**Objetivo.:**

Avaliar o conhecimento sobre ostomia em relação ao nível dos métodos de aprendizagem e mensurar a autoconfiança, a satisfação e a autoeficácia dos alunos com o treinamento recebido usando diferentes métodos de aprendizagem, incluindo simulação.

**Métodos.:**

Um estudo quase experimental de três braços com delineamento pré-teste e pós-teste em alunos do terceiro ano de enfermagem durante o ano acadêmico de 2022-2023. O grupo A recebeu métodos de ensino tradicionais e simulação de baixa fidelidade, o grupo B recebeu métodos de ensino tradicionais e simulação de alta fidelidade e o grupo C recebeu métodos de aprendizagem ativa e simulação de alta fidelidade.

**Resultados.:**

Foram encontradas diferenças significativas em 14 dos 15 indicadores NOC medidos entre os grupos. Após a intervenção, a autoeficácia apresentou diferenças significativas entre os diferentes grupos (*p*<0.001). Em relação à satisfação e à autoconfiança, também foram observadas diferenças significativas (*p*<0.001).

**Conclusões.:**

Este estudo descobriu que a simulação melhorou o nível de conhecimento sobre ostomias e teve um impacto positivo na satisfação e autoconfiança em todos os três grupos de intervenção, com resultados significativamente maiores entre os participantes do Grupo C (métodos de aprendizagem ativa e simulação de alta fidelidade).

## Introduction

An intestinal stoma is a surgical opening created in the abdominal wall to allow the passage of faeces from the intestine to an external collecting device, bypassing the usual route through the rectum and anus. Many diseases lead to its creation; however, colorectal cancer, the third most diagnosed cancer worldwide^1^ is the main cause.^2^ People with ostomies experience changes that result in impaired body image, depression, social isolation, etc.,^3^ often requiring educational interventions to address them. The ability to perform stoma care can provide the competence of the ostomate to feel confident in the management of the ostomy and improve their quality of life. The stoma care nurses are the main provider of care to these people, offering them strategies to address their new reality, ensure continuity of care, minimize complications, and reduce costs and hospitalization time, among others.^2^ Therefore, it is essential to integrate stoma care training as a fundamental competency for nursing students to ensure optimal care and support their professional, social, and familial adaptation. 

Currently, there is a significant change occurring in the clinical learning experiences of graduate nursing students. However, stoma care training still relies largely on physical manikins^4^ more than on active learning methods (ALMs), such as flipped classroom or virtual reality (VR). Among the major challenges posed by the European Higher Education Area ALMs stand out, where the student is the protagonist of his or her learning, adopting an active, creative, and autonomous role.^5^ Integrating ALMs with traditional approaches in undergraduate nursing enhances the application of theoretical knowledge in clinical practice.^6^ For their part, Information Technologies have replaced traditional teaching methods with new digital learning spaces, thereby helping in the integration of ALMs into teaching processes. Their enhances student motivation by achieving attractive, fun, and meaningful learning, ultimately improving the quality of the teaching-learning process.^7^ These techno-methodological changes have promoted new forms of knowledge transmission with great educational potential.^8^ High-fidelity simulation, like augmented and VR are considered innovative and improve academic results.^9^^,^^10^ VR allows clinical and decision-making skills to be practiced in a safe and controlled learning environment avoiding patient safety issues and ethical concerns.^11^ The use of ALMs in nursing has been associated with the development of key student competencies, such as Evidence-Based Practice^12^ or perceived clinical and cultural competencies,^13^ all of which are essential to professional practice. To date, no studies have assessed the impact of ALMs and VR simulation on knowledge, self-confidence, satisfaction, and self-efficacy in the context of ostomy care education. Thus, the results of this study could shed light on future educational modes for developing clinical competencies in ostomy care-related content. Therefore, the aims of this study are a) to assess knowledge about stoma care regarding level of learning methods; and b) to measure students' self-efficacy, satisfaction and self-confidence with the training received from the level of fidelity, including simulation. We propose the following hypothesis: (H1) The use of active learning and simulation methods with virtual reality improves the knowledge, self-efficacy, satisfaction and self-confidence of nursing students in the care of people with ostomies compared to traditional methods.

## Methods

*Study design and setting.* A quasi-experimental study of three arms was conducted to evaluate the knowledge about stoma care, the self-efficacy, and satisfaction-self-confidence with the use of different learning methods and different levels of simulation in nursing students. Group A (GA) included traditional teaching methods and low-fidelity simulation, group B (GB), included traditional teaching methods and high-fidelity simulation (HFS) and group C (GC), included active learning methods (ALMs) and HFS. The data collection period was from November to December 2022, at two different times, one week apart. The study design and procedures conform to the criteria of the "CONSORT - Simulation-Based Research Extension”.

*Participants.* A convenience sample comprised of 429 third-year nursing students enrolled in two Spanish Nursing Faculties of two public universities (academic year 2022-2023) taking the subject "Adult Nursing III" at the University of Granada (UGR) (GC and GA) and the subject "Clinical Nursing III" at the University of Jaén (UJA) (GB), all of them taught in the third year of the fifth semester. In these courses, the content of the training in stoma care is taught in small groups of 15-20 students each. 

**Figure 1:**
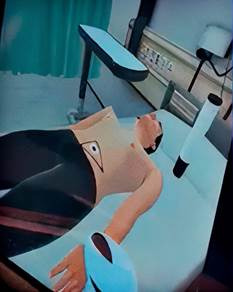
Screenshot showing a digital content of the sVR-SSIMAT

The groups within the UGR (GA and GC) were randomly assigned to each campus in such a way as to compensate for low-fidelity and high-fidelity, morning and afternoon shift. Subsequently, the third arm was (GB) included with the UJA students, being a single group. For all, Inclusion criteria were: third-year nursing students enrolled in the “Adult Nursing III" at the UGR and the subject "Clinical Nursing III" at the UJA. Exclusion criteria were students who did not participate and those who have a health problem (such as dizziness) that prevents them from wearing virtual reality glasses. Thus, GA (*n*=114 participants) and GC (*n*=165 participants) (both at the University of Granada-Granada, Ceuta, and Melilla Campuses) and GB was composed of students from the University of Jaén (*n*=83 participants). The final participation rate was 84.38% (*n*=362). The data collected were used for research purposes only and were kept in the custody of the researchers. The scenario of the *Virtual Reality stoma site marking training (sVR-SSIMAT).* The design of the sVR-SSIMAT was created by the researchers and a stomatherapy nurse based on the literature.^13^ CREAME software was used in the development of the idea. The experience immerses the student, through the Oculus Quest 2 Virtual Reality goggles. The student can see a clinical room with a patient and a tablet with the steps for a stoma marking procedure. Once the student reads the first steps about location, ribs, scars, etc., the virtual patient lies down and with a pencil the student must mark the stoma. Once the bisector point is drawn, the trainee must mark the stoma in multiple potential sites in different abdominal quadrants (Figure 1).

*Implementation phase.* For the implementation phase, three intervention groups were conducted: the intervention for GA and GC was carried out at University of Granada with a total of 23 sessions of 120 min each. The GB intervention was carried out at the University of Jaén with a total of 10 sessions lasting 180 min each. All the monitors who participated in the experience received previous training for the type of methodology to be developed. At the UGR (Granada and Ceuta Campuses) the interventions were developed by the same researcher (in addition to the teachers of the subject) while at the Melilla Campus and at the UJA, the interventions were carried out by two researchers participating in the study, who also have the role of teacher. A planning was prepared for each group of simulation (Table 1).

**Table 1:** Planning of each group of simulation

Group	Planification	Min
A: traditional teaching methods and low-fidelity simulation	Introduction of the lecturer and explanation of the content of the seminar by means of a master class.	5
	Teacher's demonstration of the contents through lecture: 1) Concept of ostomy and types of pouching; 2) General complications; 3) Peristomal skin measurement scales and marking.	45
	Students will divide the class into 4-5 groups of 4-5 people and perform teacher-guided group practice on the manikin: device change, irrigation and marking.	70
B: traditional teaching methods and high-fidelity simulation	Introduction of the lecturer and explanation of the content of the seminar by means of a master class.	45
	Teacher demonstration of the procedures to be performed: 1) Placement and removal of devices + hygiene; 2) Peristomal skin assessment with DET scale; 3) Stoma marking.	15
	Organization of the group in 3 scenarios: 1) Placement and removal of devices + hygiene; 2) Evaluation of peristomal skin with DET scale through imaging; 4) Stoma marking with virtual reality. sVR-SSIMAT.	60
C: active learning methods and high-fidelity simulation.	Introduction of the lecturer and explanation of the seminar contents.	5
	Resolution of doubts that may have arisen after consulting PRADO infographics.	15
	Individual game dynamics through 10 knowledge questions with four alternative answers that students will answer through the Socrative platform. It is a space race where, the more questions they get right, the better place they will obtain in the competition.	15
	Students will divide the class into 4-5 groups of 4-5 people and will perform group practices guided by the teacher on the manikin: changing the device, irrigation. Stoma marking with virtual reality sVR-SSIMAT (Parallel session with another teacher).	85

*Data collection procedure.* Data were collected through a questionnaire with demographic information, and the following evaluative tools: The Nursing Outcomes Classification (NOC) "Knowledge: Ostomy Care [1829]”^15^ and its indicators, self-efficacy^16^ and student satisfaction and self-confidence with learning^17^ By completing the questionnaires, participants gave their consent to participate in the study. The students were also clearly informed that their answers would not be used to evaluate them in the course. The pretest included: (i) *Questionnaire with demographic information*: It included the age of the participants, gender, class shift (morning-afternoon), university of belonging and number of times the course was taken; (ii) *Ad hoc knowledge test on nursing stoma care*: to measure the level of knowledge about stoma care, no validated scale was found. Therefore, it was considered appropriate to use the NOC outcomes "Knowledge: Ostomy Care [1829]”^15^ (Extent of understanding conveyed about maintenance of an ostomy for elimination) and its indicators. It consists of 16 indicators, observable and quantifiable measures used to evaluate the level of knowledge about ostomy care through a Likert scale from 1 to 5, where 1= No knowledge and 5=Extensive knowledge. The NOC outcomes are a standardised nursing language that has been translated into nine languages to explore and describe nursing practice in a variety of specialisms, patient groups, healthcare settings^15^ and studies to evaluate knowledge;^18^ (iii)*Self efficacy scale*: the "General Scale of Self-Efficacy",^16^ validated for the Spanish university population, was used to evaluate the student's perception of self-efficacy with respect to learning. It aims to assess the stable feeling of personal competence to effectively handle a wide variety of stressful situations. It has good internal consistency (Cronbach's alpha = 0.87) and a high validity rate. It consists of 10 items evaluated with Likert scale from 1 to 10, whose total score varies between 10 and 100 points (higher scores indicate higher levels of perceived self-efficacy). In the post-test, on the one hand, the instruments of the pre-test (excluding the demographic variables) were measured, as well as the (iv) *Satisfaction and self-confidence of the students in learning with respect to the simulation.* For this purpose, the satisfaction and self-confidence scale adapted and validated for implementation in the Spanish educational context was used. This tool evaluates clinical learning based on simulation in the Nursing degree. It has a high internal consistency (Cronbach's alpha α = 0.93) and consists of 13 items distributed in two dimensions (satisfaction/5 items and self-confidence in learning/8 items) that are evaluated with Likert scale from 1 to 5, where 1 = Totally disagree and 5 = Totally agree. This instrument was distributed to all students after the session*.*

*Data analysis.* The analysis of the data was conducted using SPSS (Statistical Package for the Social Sciences) version 28.0.1.0. Data from the descriptive analysis were presented as percentages for categorical variables and as the mean (M) along with the standard deviation (SD) for continuous variables. Different non-parametric tests were employed for inferential analysis due to the non-normality and lack of homoscedasticity, as demonstrated by the Kolmogorov-Smirnov and Levene's tests, respectively. Kruskal-Wallis tests were conducted to check for differences among fidelity groups and groups based on shifts for the variables NOC outcomes, confidence, satisfaction, and self-efficacy. Additionally, Friedman tests were carried out to compare pre- and post-simulation differences in the variables NOC outcomes and self-efficacy. Significant differences were considered for p-values less than 0.05. Students with incomplete tests were excluded from the analysis.

*Ethical considerations.* The study was approved by the Research Ethics Committee at the University of Granada (registry number: 2110/CEIH/2021) and Research Ethics Committee at the University of Jaén (registry number: OCT.22/4. PRY). Students were informed about the main goal of the investigation, and their consent was obtained digitally upon the distribution of the questionnaires. 

## Results

Participants´ characteristics*.* The sample consisted of a total of 362 undergraduates nursing students, of whom 65 (17.96%) were male, 295 (81.49%) were female, and 2 (0.55%) preferred not to disclose their gender. The average age was 22.31 years (SD = 4.98). Among the participants, 98.62% were taking the adult III subject for the first time, while the remaining had repeated the course on one or more occasions 

**Figure 2:**
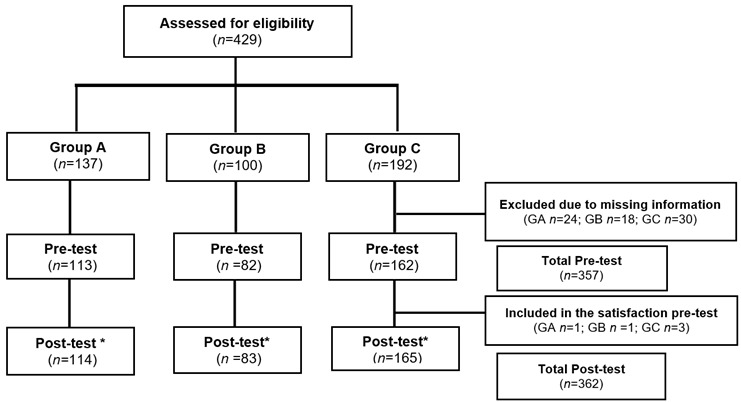
Participants´ Flow Chart

*Evaluative Tools.* To achieve the proposed objectives the different evaluative tools were completed before (pre-test) and after (post-test) the class (Figure 2). 

* Post-test was completed for more students. Its analysis was done independently for those who did not completed pre-test **Note:** Group A: traditional teaching methods and low-fidelity simulation; Group B: traditional teaching methods and high-fidelity simulation; Group C: active learning methods and high-fidelity simulation.

*The NOC indicators.* Before the simulation, no significant differences among the fidelity groups were observed in 9 out of 15 indicators. However, statistically significant differences were found after the simulation (*p*<0.001). Specifically, in 14 out of 15 NOC indicators measured (no significant differences for the "Indicator [182909]: Supplies required to care for ostomy") (Table 2). In addition to the Kruskal-Wallis analysis, a Mann-Whitney test was performed, revealing significant differences in all indicators between GA and GC, except for the indicator [182909]. In contrast, when comparing the GB and GC groups, only two indicators showed significant differences: [182911] and [182912]. Moreover, during the post hoc analysis between the GA and GB groups, significant differences were observed in 11 of the indicators measured, with the exceptions of [182902], [182908], [182909], and [182916]. 

**Table 2:** Comparison of NOC indicators pre and post-test mean scores, test statistics, and *p*-values among different learning methodologies and different levels of simulation in nursing students by group

NOC	Group A		Group B		Group C			***p-*value**	
	M± SD		M± SD		M± SD				
	Pre-test	Post-test	Pre-test	Post-test	Pre-test	Post-test	Pre-test	Post-test
[182901] Function of ostomy	2.60± 0.88	4.43 ± 0.58	2.38 ± 0.96	4.28 ± 0.64	2.58 ± 0.93	4.45 ± 0.58	0.167	<0.001
[182902] Purpose of ostomy	3.27 ± 0.94	4.62 ± 0.53	3.11 ± 1.02	4.62 ± 0.56	3.24 ± 1.03	4.66 ± 0.54	0.447	<0.001
[182903] Skin care needs around ostomy	2.63 ± 0.96	4.42 ± 0.63	2.54 ± 1.04	4.54 ± 0.57	2.76 ± 1.04	4.50 ± 0.65	0.102	<0.001
[182904] Irrigation techniques	1.80 ± 0.86	4.05 ± 0.76	1.90 ± 1.12	3.86 ± 0.67	1.88 ± 0.99	4.18 ± 0.65	0.927	<0.001
[182905] How to measure stoma	2.03 ± 1.16	4.78 ± 0.50	1.73 ± 1.10	4.65 ± 0.57	1.96 ± 1.19	4.65 ± 0.68	0.130	<0.001
[182907] Complications related to stoma	2.34 ± 0.93	4.36 ± 0.71	2.10 ± 0.90	4.29 ± 0.56	2.31 ± 1.07	4.39 ± 0.75	0.221	<0.001
[182908] Schedule for changing ostomy bag	2.35 ± 1.14	4.55 ± 0.63	1.95 ± 1.02	4.59 ± 0.56	2.29 ± 1.19	4.64 ± 0.60	0.035	<0.001
[182909] Supplies required to care for ostomy	2.45 ± 1.09	4.57 ± 0.59	2.16 ± 1.09	4.56 ± 0.58	2.29 ± 1.10	4.55 ± 0.60	0.149	0.215
[182910] Flatus-producing foods	2.75 ± 1.13	4.18 ± 0.83	2.32 ± 1.05	3.99 ± 0.66	2.70 ± 1.17	4.47 ± 0.67	0.012	<0.001
[182911] Diet modifications	2.51 ± 1.02	4.19 ± 0.83	1.96 ± 0.82	3.95 ± 0.74	2.49 ± 1.11	4.46 ± 0.64	0.001	<0.001
[182912] Fluid intake requirements	2.41 ± 1.04	4.32 ± 0.78	2.05 ± 0.91	4,07 ± 0.73	2.45 ± 1.07	4.53 ± 0.59	0.012	<0.001
[182914] Modification of daily activities	2.39 ± 0.99	4.25 ± 0.71	1.94 ± 0.81	4.18 ± 0.66	2.49 ± 1.01	4.41 ± 0.72	0.001	<0.001
[182915] Procedure to change ostomy bag	2.30 ± 1.16	4.71 ± 0.50	2.12 ± 1.17	4.62 ± 0.57	2.40 ± 1.19	4.63 ± 0.59	0.178	<0.001
[182916] Procedure to empty ostomy bag	2.12 ± 1.07	4.59 ± 0.59	2.02 ± 1.03	4.60 ± 0.61	2.22 ± 1.11	4.49 ± 0.69	0.430	<0.001
[182917] Available support groups	1.59 ± 0.77	3.95 ± 0.94	1.60 ± 0.87	3.88 ± 0.88	1.84 ± 0.96	4.12 ± 0.86	0.024	<0.001

Note: Group A: traditional teaching methods and low-fidelity simulation; Group B: traditional teaching methods and high-fidelity simulation; Group C: active learning methods and high-fidelity simulation.

A comprehensive analysis of repeated measures was performed to assess the effect of the intervention on NOC indicators responses before and after the simulation. The findings indicated a notable increase in NOC indicator scores post-simulation, showing statistically significant differences both within each group and across all three groups. These enhancements were evident after the simulation, not only in the cumulative NOC indicator scores, (Table 3) but also across all individual indicators, highlighting the impact of the intervention. 

**Table 3:** Comparison of NOC indicators in post and pre-test accumulative score among groups

	Groups			X^2^_F_	***p*-value**
	*A*	*B*	*C*		
	M± SD	M± SD	M± SD		
Pre-Intervention	35.56 ±10.83	31.88 ±10.13	36.00 ±11.34	7928	<0.001
Post-Intervention	65.96 ± 7.09	64.67 ± 6.31	67.11±7.14

Note: Group A: traditional teaching methods and low-fidelity simulation; Group B: traditional teaching methods and high-fidelity simulation; Group C: active learning methods and high-fidelity simulation.

*Self-efficacy.* Prior to the intervention, baseline self-efficacy scores did not differ significantly among the groups (*p*>0.05). This also happened with the post-intervention results as indicated by the Kruskal-Wallis test, although a tendency toward higher scores was noted in the GC Table 4). The analysis between fidelity groups (Mann-Whitney test) revealed no differences when comparing the groups pairwise (either in the pre- or post-simulation results). Nevertheless, when analysing the changes in self-efficacy scores after the intervention (pre- and post-simulation), a significant overall difference was identified among all the participant groups (*p*<0.001).

**Table 4:** Comparison of self-efficacy final score in post-test and pre-test (individually) and differences between pre- and post-intervention scores

Moment	Group A	Group B	Group C	**H/*p*-value**	X^2^_F/_ *p*-value*
	M± SD	M± SD	M± SD		
Pre-Intervention	70.76 ±1 2.70	69.66 ± 11.62	73.85 ± 10.94	5.79 / 0.055	188.478 / <0.001
Post-Intervention	77.94 ± 14.07	78.06 ± 12.72	81.28 ± 10.76	3.267 / 0.195

*H= Kruskal-Wallis test; X^2^_F_= Friedman test

Note: Group A: traditional teaching methods and low-fidelity simulation; Group B: traditional teaching methods and high-fidelity simulation; Group C: active learning methods and high-fidelity simulation.

*Student satisfaction and self-confidence.* Regarding learning satisfaction and self-confidence, the intervention revealed significant differences across the groups. The satisfaction scores reported in GC were higher than GA (Table 5). Likewise, self-confidence scores exhibited significant variations among the groups. Notably, the GC reported higher self-confidence levels compared to GA. Comparing satisfaction and self-confidence scores (Mann-Whitney test) among the fidelity groups pairwise, differences were observed between GA and GB/GC, and GB and GC (*p*<0.001). 

**Table 5:** Comparison of satisfaction and self-confidence among groups

Variables	*Group A*	*Group B*	*Group C*	H*	***p*-value**
	M± SD	M± SD	M± SD		
Satisfaction	21.69 ± 4.36	24.76 ± 2.22	22.99 ± 4.512	74.046	<0.001
Self-confidence	33.50 ± 6.38	39.92 ± 0.28	34.44 ± 7.28	115.85	<0.001
*H= Kruskal-Wallis test

Note: Group A: traditional teaching methods and low-fidelity simulation; Group B: traditional teaching methods and high-fidelity simulation; Group C: active learning methods and high-fidelity simulation.

## Discussion

This study addresses the differences in learning methods as well as the level of knowledge, self-efficacy, satisfaction, and self-confidence of nursing students in stoma care depending on the level of fidelity of the simulation.

*The NOC outcome “Knowledge: Ostomy Care [1829]”.* In this study, the simulation appeared to positively influence the cognitive ability of nursing students, indicating an improvement in the acquisition of knowledge for the care of people carrying digestive stoma. The results point to an improvement in knowledge in the GC and GB groups, compared to the GA group. These results are consistent with other studies that show the use of high-fidelity simulation in nursing education is more effective than the traditional method in terms of knowledge improvement, skill levels for cardiac auscultation, and anxiety reduction.^19^ On the same line, Aljohani *et al*.^20^ show an improvement in training in cardiac life support with simulation. For their part, So *et al.*^21^ find that simulation-based learning increases the retentive ability of nursing students by improving their future learning skills. Finally, other authors argue that the integration of ALMs with simulation technology increases the acquisition of knowledge.^22^

*Self-efficacy.* Although self-efficacy in this study did not present significant differences between the different intervention groups, the result is consistent with other authors in which no improvements were found when comparing the simulation with the traditional methodology.^23^ This may be because, as some papers report,^24^, self-efficacy declines significantly as students’ progress in academic years. These results suggest that simulation may not have a uniform impact on students' self-efficacy and that other factors may influence these results. Thus, it is important to explore other variables that may affect the perception of self-efficacy of university students in relation to simulation due to the effects it may have on the development of adaptive academic goals and successful effort in nursing students.^25^ Since self-efficacy is a complex construct and depends on several factors, such as mastery experience, repeated success in tasks could increase positive self-efficacy in self-assessments whereas repeated failures could decrease them.^26^ Therefore, considering that the students only had one opportunity to experience VR, the absence of significant differences in self-efficacy between groups could be attributed to this limited practice.

*Satisfaction and self-confidence. R*egarding the level of satisfaction and self-confidence of the students, the GB and GC achieved higher scores than the GA. This suggests that HFS increases the satisfaction and self-confidence of nursing students in stoma care. So, Zapko *et al*.^27^ conclude that using simulations of patients with HFS and MFD, and actors as patients in different real scenarios (e.g., mental health, critical care, obstetrics, medical/surgical, and geriatric scenarios), students improved satisfaction and confidence. Another study^28^ examines the use of simulation for cardiopulmonary resuscitation learning in nursing students, revealing high satisfaction and self-confidence scores. Meanwhile, Tawalbeh^29^ studied the effect of simulation on the learning of nursing care in critical patients with cardiac, respiratory, and neurological problems, revealing an increase in student self-confidence after the simulation. So *et al*.^21^ describe that the increase in student self-confidence is due to the simulation providing a safe environment where students can apply care without fear of harming patients, which positively influences satisfaction, contributing to the development of self-confidence and essential skills to provide safe care to patients. 

Despite the results, the inclusion of ALMs in higher education classrooms still requires ongoing training and continuing professional development^30^ as well as investment in ALMs training courses by the educational institution^31^ to enable further research in this regard. The use of VR in health sciences education can offer significant benefits, such as enhanced engagement students as well as demonstrating comparable effectiveness to standards of care,^32^ but it must be regulated, as it presents several risks. It may hinder critical thinking by promoting passive content acceptance and can cause physical issues like motion sickness, eye strain, and fatigue. Additionally, heightened immersion may lead to cognitive effects such as stress and disorientation.^33^

Conclusions. It has been found that simulation improves the level of knowledge in relation to ostomies as well as that the degree of fidelity of simulation has a positive impact on student satisfaction and self-confidence in learning. These findings offer an opportunity to implement ALM in stoma care teaching and highlight the need to validate scales to measure the knowledge about stoma care. This will help in future studies to measure more properly active learning methods in the topic, helping teachers to evaluate the simulation, enabling them to improve it. 

Limitations. There are several limiting factors in this research. First, the study was conducted across two different universities and four campuses to obtain a considerable sample, which may have introduced potential bias. Conducting the same study at a single university could help reduce this bias. Additionally, the involvement of different researchers in guiding the experience at each campus may have introduced variability in how the intervention was carried out, impacting consistency. Regarding the measurement instruments, the lack of validated scales to assess knowledge of ostomies in nursing students led to the use of NOC outcomes and indicators, emphasizing the need for validated tools in this area. Furthermore, the gender imbalance, with the nursing degree being predominantly female, prevented an analysis of this category. Lastly, the limited existing literature on simulation in the area of ostomies, particularly with VR, meant that the data could not be compared with similar experiences, further limiting the contextual analysis.
